# Protein-protein interaction prediction using a hybrid feature representation and a stacked generalization scheme

**DOI:** 10.1186/s12859-019-2907-1

**Published:** 2019-06-10

**Authors:** Kuan-Hsi Chen, Tsai-Feng Wang, Yuh-Jyh Hu

**Affiliations:** 1 College of Computer Science, National Chiao Tung University, Hsinchu, 300 Taiwan; 2 Institute of Data Science and Engineering, National Chiao Tung University, Hsinchu, 300 Taiwan; 3 Institute of Biomedical Engineering, College of Computer Science, National Chiao Tung University, Hsinchu, 300 Taiwan

**Keywords:** Protein-protein interaction, Stacked generalization, Gene ontology, Network topology

## Abstract

**Background:**

Although various machine learning-based predictors have been developed for estimating protein–protein interactions, their performances vary with dataset and species, and are affected by two primary aspects: choice of learning algorithm, and the representation of protein pairs. To improve the performance of predicting protein–protein interactions, we exploit the synergy of multiple learning algorithms, and utilize the expressiveness of different protein-pair features.

**Results:**

We developed a stacked generalization scheme that integrates five learning algorithms. We also designed three types of protein-pair features based on the physicochemical properties of amino acids, gene ontology annotations, and interaction network topologies. When tested on 19 published datasets collected from eight species, the proposed approach achieved a significantly higher or comparable overall performance, compared with seven competitive predictors.

**Conclusion:**

We introduced an ensemble learning approach for PPI prediction that integrated multiple learning algorithms and different protein-pair representations. The extensive comparisons with other state-of-the-art prediction tools demonstrated the feasibility and superiority of the proposed method.

## Background

Cells are predominantly composed of proteins, and almost every primary cellular process is performed by multiprotein complexes. By identifying and analyzing the components of protein complexes, we can better understand how protein ensembles are organized into functional units [1]. As protein–protein interactions (PPIs) are crucial to most cellular functions, they must be identified for deciphering cellular behaviors. In the past few decades, large-scale PPI analysis has been enabled by techniques such as yeast two-hybrid (Y2H) systems [2], mass spectrometry [3], and protein chips [4]. However, these methods are time-consuming and expensive, and large-scale experiments usually suffer from high false positive rates [5]. Meanwhile, computational techniques can identify potential PPIs that are not discoverable by high-throughput methods. The computational predictions can then be verified by more labor-intensive methods.

Researchers have proposed different types of computational approaches based on different sources of biological information. For example, several methods can predict PPIs from protein sequences. SPRINT evaluates the likelihood of interactions by assessing the contributions of similar sequence motifs [6]. Huang et al. [7] translated protein sequences into feature vectors of composition and transition descriptors, and predicted the PPIs using a weighted sparse representation-based classifier. Guo et al. [8] combined a support vector machine (SVM) with auto covariance that predicts PPIs from protein sequences. Other methods utilize the genomic, proteomic, and/or structural information of proteins [9, 10]. In recent years, semantic similarity has been applied to ontology, providing a valuable indicator of the relatedness level between two biological entities [11]. Observationally, proteins will likely interact when localized in the same cellular component, or when sharing a common biological process or molecular function. Accordingly, various methods infer PPIs from the gene ontology (GO) annotations and semantic similarity of proteins [12–14]. Other methods integrate semantic similarity with machine learning (ML) algorithms. For example, Ben-Hur and Noble [15], Bandyopadhyay and Mallick [16], and Armean et al. [17] combined GO annotations with SVM for PPI prediction. Other ML algorithms employed in PPI prediction include Bayesian classifiers [18] and random forest (RF) [19]. In addition, deep learning has recently been applied for PPI prediction. Sun et al. [20] used stacked autoencoders in their network architecture, Du et al. [21] adopted two separate deep neural networks to process the characteristics of each protein in a protein pair, and Gonzalez-Lopez et al. [22] introduced a deep recurrent neural network combined with the embedding techniques. These computational methods differ in their feature representations and algorithmic processes. Different ML approaches have distinctive inherent biases, including representation biases and process biases, which affect their learning behaviors and performances significantly even in the same learning task [23].

In this study, we propose a hybrid feature representation that combines protein sequence properties, gene ontology information, and interaction network topology. To reflect the characteristics of amino acids, we encode their various physicochemical properties (such as hydrophobicity, hydrophilicity, polarity and solvent accessible surface area) into the sequence-based features. To learn the knowledge organized in a directed acyclic graph (DAG) from GO, we develop the GO-based features by clustering the GO terms based on the partitioning of the GO DAG with respect to the provided training data. To address PPI prediction using a network reconstruction problem, we construct a partial network from the training data, and extract its topological properties as the network-based features. We adopt a stacked generalization scheme [24] and develop a classifier called PPI-MetaGO, which improves PPI prediction by deducing the biases of the base generalizers and exploiting the synergy among various ML algorithms.

PPI-MetaGo was evaluated in consistent and unbiased tests on the datasets used in previous evaluations of state-of-the-art PPI prediction methods. The experimental results demonstrate the superior performance of PPI-MetaGO over several established PPI-prediction approaches.

## Methods

**Fig. 1:**
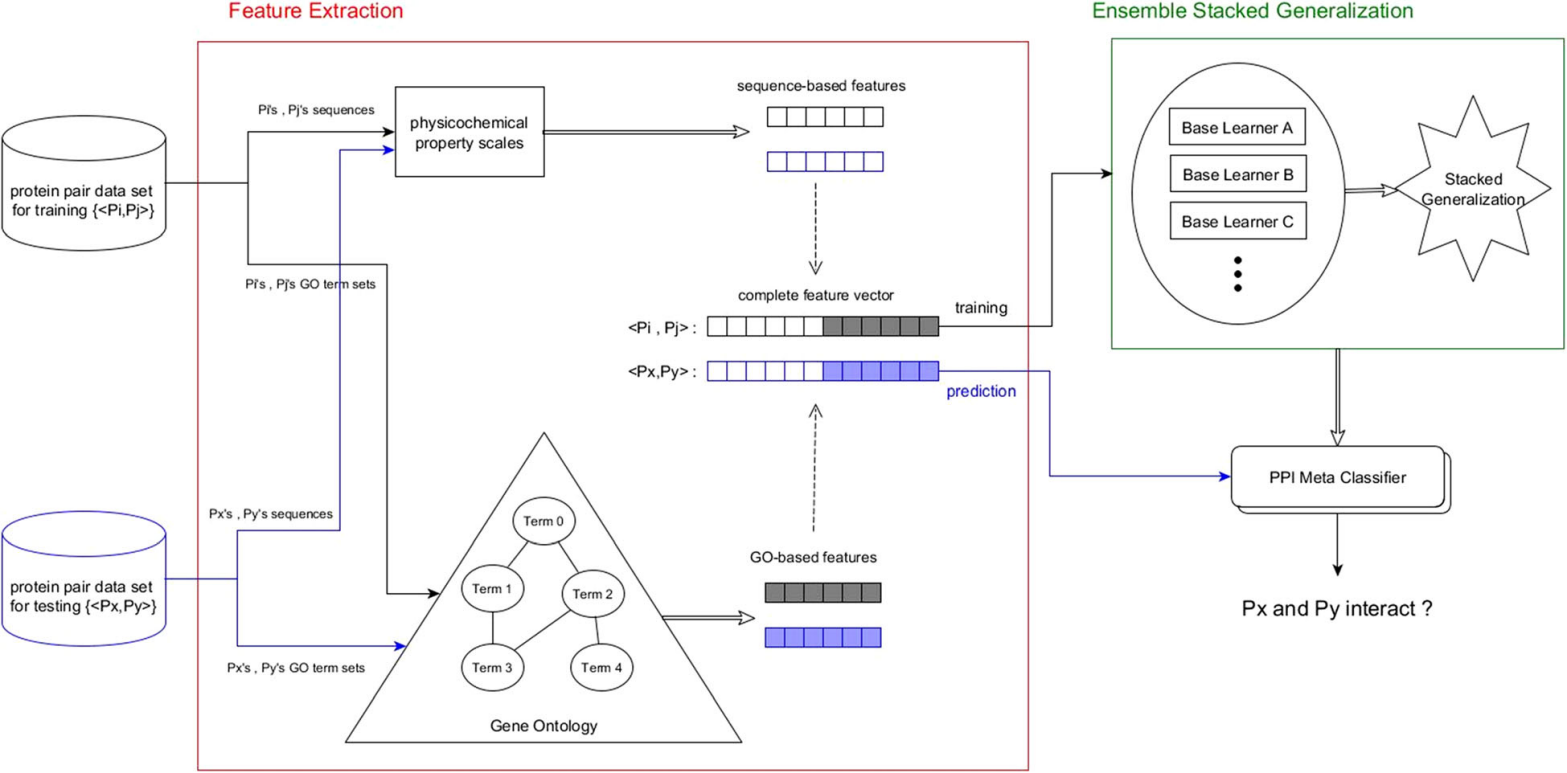
Architecture of PPI-MetaGO for predicting protein–protein interactions

This section describes our proposed ensemble supervised meta-learner PPI-MetaGO for PPI prediction. The protein pairs for training the ensemble meta-learner are represented in feature vectors constructed from the sequence-based physicochemical properties and the GO-based semantic similarities. The PPI-MetaGO is implemented as illustrated in Fig. 1.

### Feature extraction: sequence-based physicochemical features

**Fig. 2:**
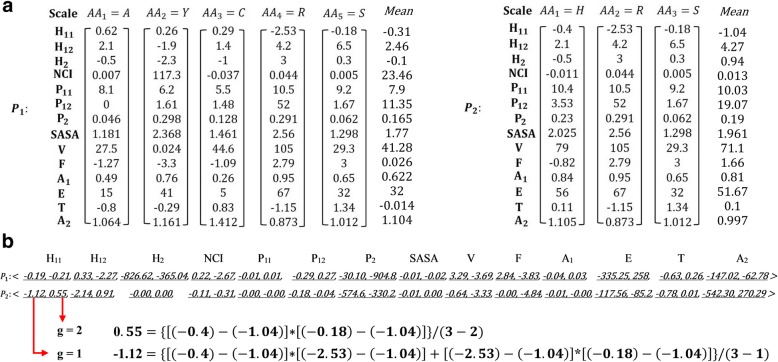
Vectorial representations of two proteins, *P*_*1*_ and *P*_*2*_. **a** Each amino acid *AA*_*i*_ is first translated into a vector of 14 physicochemical scale values, **b** Both proteins, *P*_*1*_ and *P*_*2*_, are later represented in a uniform vectorial form with 28 AC values. We demonstrate the calculation of the first two AC values of H_11_ for *P*_*2*_ when the gap is 1 (g = 1) and 2 (g = 2), respectively

As the basis for PPI prediction, we characterize proteins by 12 physicochemical properties of their composite amino acids [25–32], namely, hydrophilicity, flexibility, accessibility, turns scale, exposed surface, polarity, antegenic propensity, hydrophobicity, net charge index of the side chains, polarizability, solvent accessible surface area, and side-chain volume. Among the 12 properties, hydrophobicity and polarity are each calculated according to two different scales, respectively. The values of 14 physicochemical property scales of the 20 essential amino acids are listed in Table 1. We translated each amino acid into a vector of 14 numeric values, each corresponding to a physicochemical scale value in Table 1. As an example, Fig. 2(a) shows the transformation of two proteins, *P*_*1*_ and *P*_*2*_, into 14-element vectors. Each element in each vector corresponds to a physicochemical scale value [20, 33].

**Table 1:** Values of the 14 physicochemical property scales of the 20 essential amino acids

AA	H_11_^a^	H_12_^a^	H_2_	NCI	P_11_^a^	P_12_^a^	P_2_	SASA	V	F	A_1_	E	T	A_2_
A	0.62	2.1	− 0.5	0.007	8.1	0	0.046	1.181	27.5	−1.27	0.49	15	−0.8	1.064
C	0.29	1.4	−1.0	−0.037	5.5	1.48	0.128	1.461	44.6	−1.09	0.26	5	0.83	1.412
D	−0.9	10.0	3.0	−0.024	13.0	40.7	0.105	1.587	40.0	1.42	0.78	50	1.65	0.866
E	−0.74	7.8	3.0	0.007	12.3	49.91	0.151	1.862	62.0	1.6	0.84	55	−0.92	0.851
F	1.19	−9.2	−2.5	0.038	5.2	0.35	0.29	2.228	115.5	−2.14	0.42	10	0.18	1.091
G	0.48	5.7	0.0	0.179	9.0	0	0	0.881	0	1.86	0.48	10	−0.55	0.874
H	−0.4	2.1	−0.5	−0.011	10.4	3.53	0.23	2.025	79.0	−0.82	0.84	56	0.11	1.105
I	1.38	−8.0	−1.8	0.022	5.2	0.15	0.186	1.81	93.5	−2.89	0.34	13	−1.53	1.152
K	−1.5	5.7	3.0	0.018	11.3	49.5	0.219	2.258	100	2.88	0.97	85	−1.06	0.93
L	1.06	−9.2	−1.8	0.052	4.9	0.45	0.186	1.931	93.5	−2.29	0.4	16	−1.01	1.25
M	0.64	−4.2	−1.3	0.003	5.7	1.43	0.221	2.034	94.1	−1.84	0.48	20	−1.48	0.826
N	−0.78	7.0	2.0	0.005	11.6	3.38	0.134	1.655	58.7	1.77	0.81	49	3.0	0.776
P	0.12	2.1	0.0	0.240	8.0	0	0.131	1.468	41.9	0.52	0.49	15	−0.8	1.064
Q	−0.85	6.0	0.2	0.049	10.5	3.53	0.18	1.932	80.7	1.18	0.84	56	0.11	1.015
R	−2.53	4.2	3.0	0.044	10.5	52.0	0.291	2.56	105	2.79	0.95	67	−1.15	0.873
S	−0.18	6.5	0.3	0.005	9.2	1.67	0.062	1.298	29.3	3.0	0.65	32	1.34	1.012
T	−0.05	5.2	−0.4	0.003	8.6	1.66	0.108	1.525	51.3	1.18	0.7	32	0.27	0.909
V	1.08	−3.7	−1.5	0.057	5.9	0.13	0.14	1.645	71.5	−1.75	0.36	14	−0.83	1.383
W	0.81	−10	−3.4	0.038	5.4	2.1	0.409	2.663	145.5	−3.78	0.51	17	−0.97	0.893
Y	0.26	−1.9	−2.3	117.3	6.2	1.61	0.298	2.368	0.024	−3.3	0.76	41	−0.29	1.161

*H*_*11*_ & *H*_*12*_ hydrophobicity, *H*_*2*_ hydrophilicity, *NCI* net charge index of side chains, *P*_*11*_ & *P*_*12*_ polarity, *P*_*2*_ polarizability, *SASA* solvent-accessible surface area, *V* volume of side chains, *F* Flexibility, *A*_*1*_ Accessibility, *E* Exposed, *T* Turns, *A*_*2*_ Antegenic

^a^Hydrophobicity (H_11_ & H_12_) and polarity (P_11_ & P_12_) were calculated by two different methods

As proteins vary widely in length, different proteins can be represented by different numbers of vectors. Meanwhile, the base classifier in an ensemble meta-learner, such as an artificial neural network (ANN), k-nearest-neighbor (KNN) or naïve Bayesian (NB), requires a uniform input. For example in Fig. 2(a), protein *P*_*1*_ composed of five amino acids is represented by five vectors, but protein *P*_*2*_ with three amino acids is described by three vectors. To prepare a uniform input for the base classifier of the ensemble meta-learner, we transform the protein representation (a set of variable numbers of numeric vectors) into a uniform vectorial form with auto covariance [8, 34], in which all proteins with varying numbers of amino acids are represented by vectors of the same length. The auto covariance (AC) of the physicochemical property scale of a protein sequence describes the average interactions between the amino acids separated by a certain gap throughout the entire protein sequence. Here, the gap is set as a certain number of residues between an amino acid and its neighbor. The AC of the *i*th physicochemical property scale, *AC*_*i,g*_, is given by

1 $$ {AC}_{i,g}=\frac{1}{L-g}{\sum}_{j=1}^{L-g}\left({P}_{i,j}-{\mu}_i\right)\times \left({P}_{i,j+g}-{\mu}_i\right) $$

2 $$ {\mu}_i=\frac{1}{L}{\sum}_{j=1}^L{P}_{i,j} $$

where *g* is the pre-specified gap, *L* is the length of protein *P*, and μ_i_ is the mean of the *i*th physicochemical scale values of protein *P*. Setting the maximum gap to *G* (i.e. *g* = 1, 2, 3, …, *G*), we can represent any protein (regardless of length) as a vector of *k* × *G* AC variables, where *k* is the number of physicochemical property scales. Using auto covariance between amino acids, we are able to process the raw physicochemical scale values into a uniform vectorial form. All proteins, regardless of their lengths, can consequently be represented by vectors of the same length. For example, when *G* is set to 2 and there are 14 physicochemical scales, the numeric vectors of proteins *P*_*1*_ and *P*_*2*_ in Fig. 2(a) can be transformed into a uniform AC vectorial form shown in Fig. 2(b). Proteins *P*_*1*_ and *P*_*2*_ are represented by a vector of 28 AC values, respectively, even though they have different lengths.

To avoid the effects of variance, we first normalize the AC of each property scale to zero mean and unit standard deviation as follows:

3 $$ {S}_i=\frac{A_i-{\mu}_i}{SD_i},i=1\cdots M,\kern0.5em $$

where *S*_*i*_ is the standardized value, *A*_*i*_ is the raw value of the *i*th AC, μ_i_ and *SD*_*i*_ denote the mean and standard deviation of the *i*th AC, respectively, and *M* is the number of AC values in the AC vector. Secondly, to ensure that the ACs derived from different physicochemical scales are commensurate and to further suppress the effects of outliers, we adopt a min–max scaling method that scales the standardized AC values to a fixed range of [0, 1]. The min–max scaling is described by Eq. (4).

4 $$ {V}_i=\frac{S_i-{\mathit{\min}}_i}{{\mathit{\operatorname{MAX}}}_i-{\mathit{\min}}_i},i=1\cdots M,\kern0.5em $$

where *V*_*i*_ is the scaled value, *S*_*i*_ is the standardized value of the *i*th AC, *MAX*_*i*_ and *min*_*i*_ are the maximum and minimum of the standardized values of the *i*th AC, respectively, and *M* is defined above.

With two proteins represented by two AC vectors, protein pair (*P*_*1*_, *P*_*2*_) can be represented in one of two common forms: (1) combination [V(*P*_*1*_) ⊕ V(*P*_*2*_)], or (2) concatenation [V(*P*_*1*_)V(*P*_*2*_)]. Here, V(*P*) is the sequence-based feature vector corresponding to protein *P*, and the ⊕ operator adds the feature values of the two proteins in element-by-element fashion [16]. In our approach, the element-by-element feature values of two proteins are combined by concatenating the feature vectors. The concatenation avoids the need for applying a direct pairwise kernel on the feature space of protein pairs [16], which involves a complex kernel design, or applying specific binary operators such as addition or multiplication to each pair of elements, which introduce uncertain effects. However as mentioned in Bandyopadhyay and Mallick [16], concatenating the protein pair features is undesirable in PPI prediction because for the same protein pair *P*_*1*_ and *P*_*2*_, [V(*P*_*1*_)V(*P*_*2*_)] and [V(*P*_*2*_)V(*P*_*1*_)] are differently represented in the feature space. Training a learner by one of the two representations will lose the information of the other representation. To resolve the order problem, we represent the protein pair (*P*_*1*_, *P*_*2*_) by both concatenations, [V(*P*_*1*_)V(*P*_*2*_)] and [V(*P*_*2*_)V(*P*_*1*_)]. Provided with the concatenations in both orders for training, the learner can flexibly identify the (approximately) optimum decision regions for the PPI prediction, based on either of [V(*P*_*1*_)V(*P*_*2*_)] or [V(*P*_*2*_)V(*P*_*1*_)]. To classify a new protein pair (*P*_*3*_, *P*_*4*_), we average the predicted class probabilities (interacting and non-interacting) produced by the trained learner for [V(*P*_*3*_)V(*P*_*4*_)] and [V(*P*_*4*_)V(*P*_*3*_)], respectively, and predict the class of the protein pair (*P*_*3*_, *P*_*4*_) according to the higher average probability.

### Feature extraction: GO-based features

GO is a hierarchical vocabulary for annotating gene functions and their relationships with respect to their molecular function (MF), cellular components (CC), and biological process (BP) [35]. Each subontology is represented by a rooted DAG, where each node corresponds to a GO-term, and each link denotes a relationship between two terms, such as *part_of* or *is_a*. This hierarchical knowledge of the functional relationships between gene products has proved most useful for assessing the relevance of the involvement of genes in various biological activities [36], including PPI prediction [13, 16, 19].

Interacting proteins often participate in similar biological processes, exercise similar molecular functions, and/or co-localize in similar cellular components; consequently, they exhibit high GO semantic similarity [14, 37]. Many measures of semantic similarity have been proposed and categorized into edge-based, node-based and hybrid methods [11]. The edge-based methods are mainly based on counting the edges along the paths between the GO terms being considered [38]. By contrast, the node-based approaches compare the properties of the involved terms, their ancestors, or their descendants [39, 40]. One of the most commonly considered properties is the information content of the terms. Node-based measures are typically more reliable than edge-based methods in the biomedical field, because most of the edge-based measures assume that the distance between all relationships in an ontology is constant or depth-dependent. Neither assumption is valid in existing biomedical ontologies. Alternatively, the hybrid methods assign weights to the edges and defines the semantic similarity after combining various types of measures, such as node depth, node link density, information content, or semantic contribution of the relationships (e.g. *is_a* or *part_of*) [41].

**Fig. 3:**
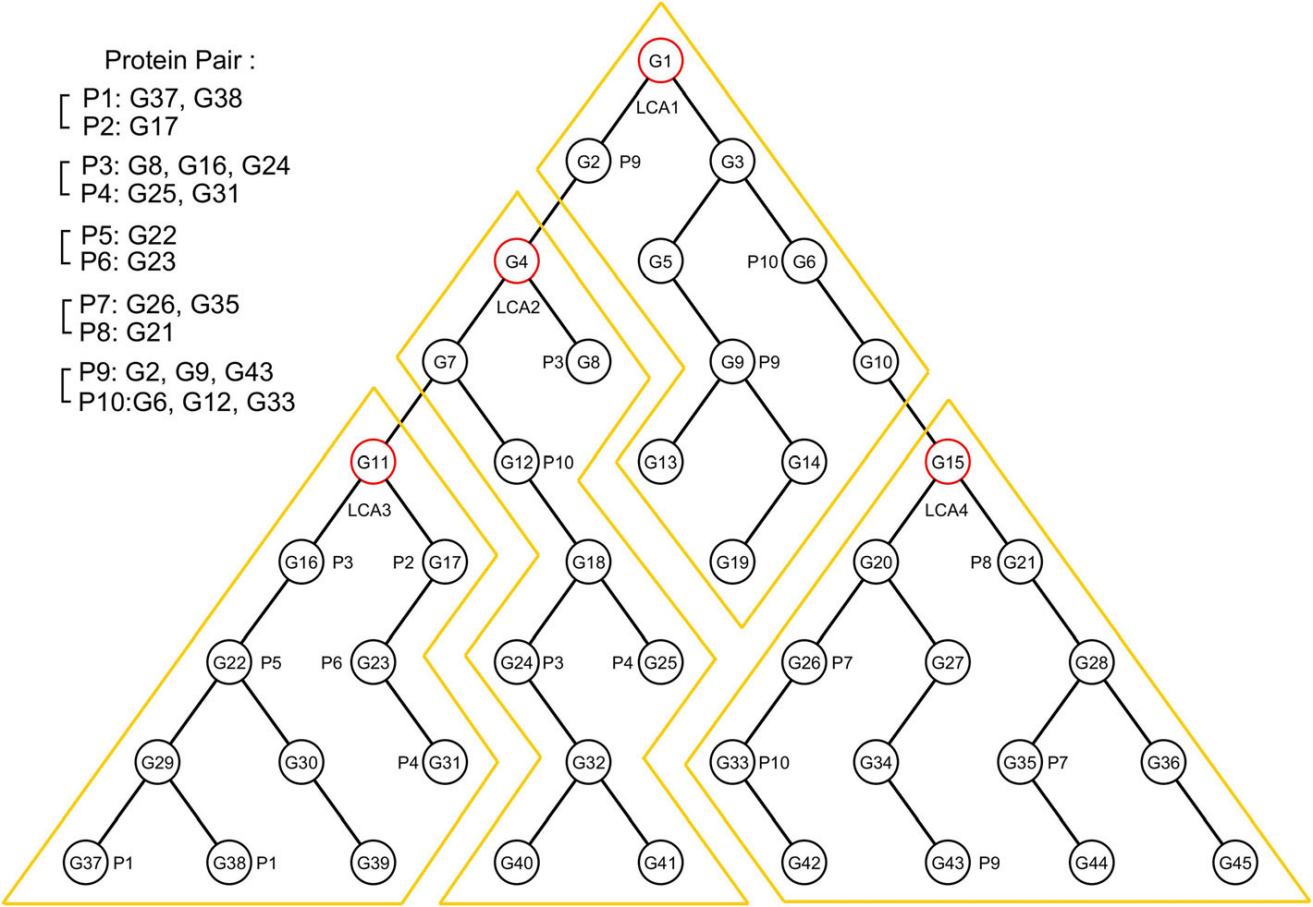
Demonstration of GO DAG partitioning into clusters based on LCAs

We propose a novel approach that characterizes protein pairs based on the clustering of GO terms. Given two sets *G*_*i*_ and *G*_*j*_ of GO terms annotating each of the proteins *P*_*i*_ and *P*_*j*_ in a pair, we traverse the GO hierarchy from the GO terms in *G*_*i*_ and *G*_*j*_ up to their lowest common ancestor (ULCA) [19]. In this fashion, we identify the lowest common ancestor (LCA) of each protein pair <*P*_*i*_*, P*_*j*_ > in a given set of protein pairs. The found LCAs are stored in a list sorted by ascending order of their hierarchical GO level. For each LCA in the sorted list in ascending order, we iteratively group that LCA and all its descendants into a cluster, excluding those already assigned to a previously formed cluster. The entire GO DAG is consequently partitioned into a set of mutually exclusive subgraphs, each rooted by an LCA, as illustrated in Fig. 3. In the sample hierarchy of Fig. 3, the two protein pairs <P_1_,P_2_ > and < P_5_,P_6_ > share a common LCA (G_11_), which is denoted by LCA_3_. The LCA of protein pair <P_3_,P_4_ > (G_4_) is denoted by LCA_2_. The LCAs of protein pairs <P_7_,P_8_ > and < P_9_,P_10_ > (G_15_ and G_1_ respectively), are denoted by LCA_4_ and LCA_1_, respectively. These four LCAs are organized into a sorted list *L* in ascending order of their hierarchical levels, namely, *L* = (LCA_4_, LCA_3_, LCA_2_, LCA_1_). The first LCA in the sorted list, LCA_4_, is grouped with all its descendants in the hierarchy. The resulting cluster contains G_15_, G_20_, G_21_, G_26_, G_27_, G_28_, G_33_, G_34_, G_35_, G_36_, G_42_, G_43_, G_44_, and G_45_. Similarly, by grouping all the descendants from G_11_ (i.e. LCA_3_), we represent the second cluster of GO terms by a hierarchical subgraph rooted at G_11_. This subgroup contains 11 GO terms, including G_11_ itself. Continuing to the next LCA in the list, LCA_2_, we cluster all descendants of G_4_ (i.e. LCA_2_) that have not been assigned to an earlier cluster. Excluding the terms included in the second cluster, we form the third cluster of GO terms, constituting G_4_, G_7_, G_8_, G_12_, G_18_, G_24_, G_25_, G_32_, G_40_ and G_41_. Finally, based on LCA_1_, we group G_1_, G_2_, G_3_, G_5_, G_6_, G_9_, G_10_, G_13_, G_14_ and G_19_ into the fourth cluster. The entire hierarchy is consequently partitioned into four subgraphs, each corresponding to an LCA, based on the provided training set of protein pairs, namely, {<*P*_*1*_*, P*_*2*_>,<*P*_*3*_*, P*_*4*_>,<*P*_*5*_*, P*_*6*_>,<*P*_*7*_*, P*_*8*_>}. Provided with different training protein pairs, we can partition the hierarchy accordingly to reflect the different interaction characteristics of the protein pairs.

**Fig. 4:**
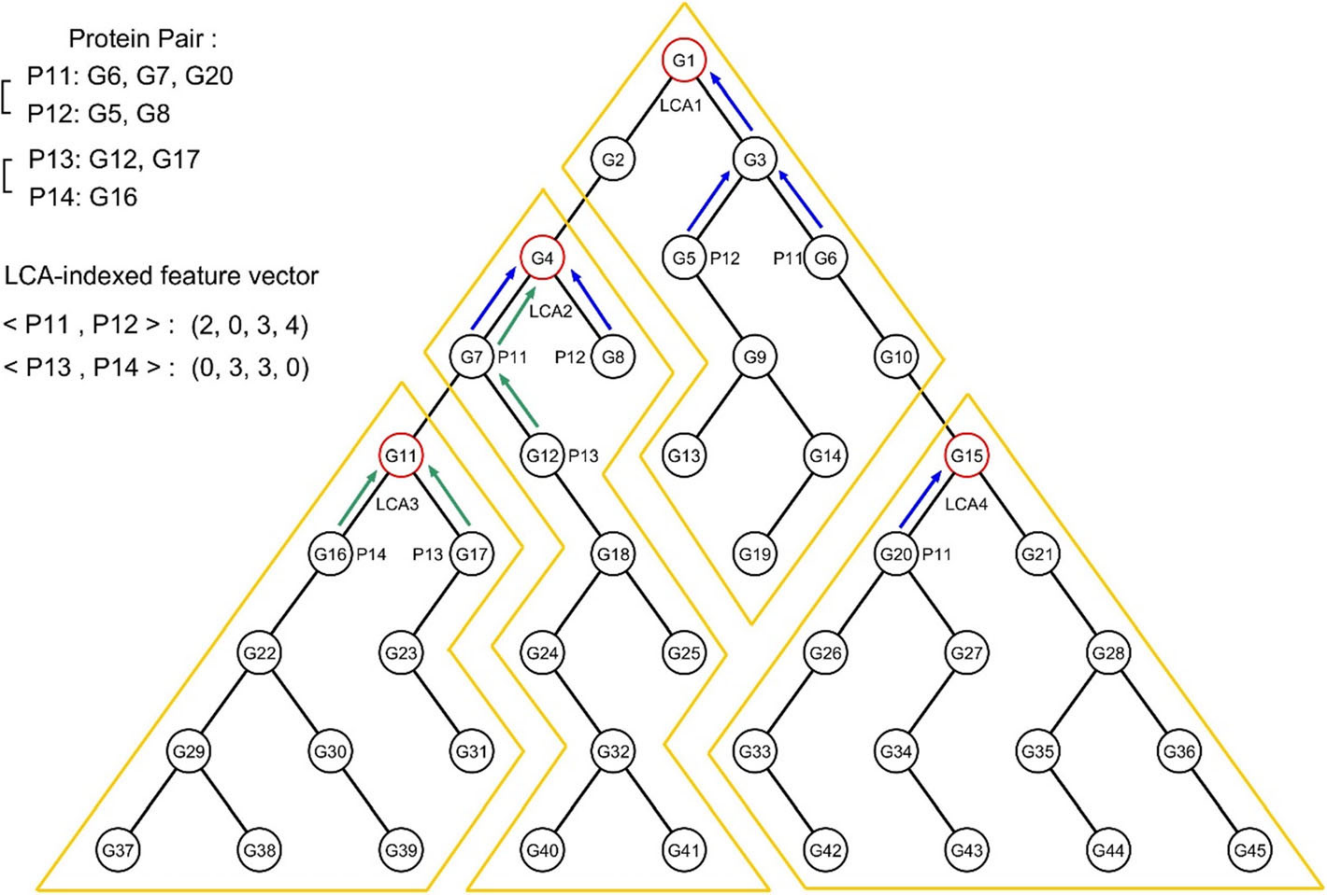
Example of encoding protein pairs into LCA-indexed GO-term feature vectors. The blue and green arrows show the ascending traversals up to the LCAs from the GO terms of <*P*_*11*_, *P*_*12*_ > and < *P*_*13*_, *P*_*14*_>, respectively

Feature vectors of GO-terms have been constructed by considering the presence or absence of shared GO terms [19], or weighting the GO terms by their information content and local topology [16]. Instead, we define one GO-based feature as one GO-term cluster indexed by an LCA. To translate the sets of GO-term annotations *G*_*i*_ and *G*_*j*_ for each protein pair <*P*_*i*_, *P*_*j*_ > into numeric values of LCA-indexed GO-based features, we first locate the GO terms in sets *G*_*i*_ and *G*_*j*_ on each LCA-indexed subgraph. For each GO-term, we count the nodes along the ascending path up to the root of a subgraph, and sum the node counts on the subgraph. This sum is assigned as the value of the corresponding GO-term feature. Figure 4 shows the encoding of two protein pairs into two feature vectors, based on the four LCA-indexed GO-term features presented in Fig. 3. To obtain the LCA-indexed GO-term feature vector for the protein pair <*P*_*11*_, *P*_*12*_>, we locate the GO terms of *P*_*i*_ and *P*_*j*_ on the hierarchy. The GO terms G_5_ and G_6_ are located in the subgraph of LCA_1_, terms G_7_ and G_8_ are located in the subgraph of LCA_2_, and G_20_ is located in the subgraph of LCA_4_. The subgraph rooted at LCA_3_ contains no GO-term of either *P*_*11*_ or *P*_*12*_. Tracing along the ascending paths from G_5_ and G_6_ up to LCA_1_ (blue arrows on the subgraph of LCA_1_ in Fig. 4), we encounter G_5_, G_6_, G_3_, and G_1_ (a total of four nodes). Therefore, the value of the LCA_1_-indexed GO-term feature is 4. Similarly, the values of the GO-term features indexed by LCA_2_ and LCA_4_ are determined as 3 and 2, respectively. As the subgraph of LCA_3_ contains no GO terms of either *P*_*11*_ or *P*_*12*_, the Go-term features indexed by LCA_3_ are assigned a value of zero. Finally, the LCA-indexed GO-term feature vector for <*P*_*11*_, *P*_*12*_ > is obtained as (2, 0, 3, 4). The GO terms of <*P*_*13*_, *P*_*14*_ > are converted into a GO-term feature vector (0, 3, 3, 0) by the same process (see Fig. 4). Because the partitioning of the GO DAG depends on the given training data, the GO-based features of the same protein pair can vary in number and their values to adapt dynamically to the changes of training data. This flexibility warrants a better definition of GO-based features and leads to higher predictive performances when the size and the quality of training data increase.

### Feature extraction: network-based features

We derive the network-based features from the topological properties of a PPI network, *N*_*PPI*_ *= <V, E>*, where *V* and *E* denote the node and link sets, respectively. Here, each node represents a protein, and each link is an interaction between two proteins. To predict the PPI of a set of proteins, we construct the PPI network *N*_*PPI*_, and whether two proteins are linked in *N*_*PPI*_ depends on the semantic similarity of their GO terms. The functional similarity between two gene products can be determined by various similarity measures, some of which were originally developed for natural language taxonomy [37, 40, 41]. We here measure the functional similarity between proteins by the widely used Resnik’s measure [40], which has proven superior in several prominent studies [12, 39, 42].

Resnik’s measure quantifies the semantic similarity between two ontology terms *t*_*i*_ and *t*_*j*_ as the information content (IC) of their most informative common ancestor (MICA) [11, 13, 40]. Resnik’s semantic similarity between *t*_*i*_ and *t*_*j*_ is defined by Eq. (5):

5 $$ {Sim}_{Resnik}\left({t}_i,{t}_j\right)=\max \left\{ IC(t)|t\in CA\left({t}_i,{t}_j\right)\right\},\kern0.5em $$

where *CA* (*t*_*i*_, *t*_*j*_) is the set of common ancestors of *t*_*i*_ and *t*_*j*_ in the GO hierarchy, and *IC*(*t*) is the information content of term *t. IC*(*t*) is defined by –log *p*(*t*), where *p*(*t*) is the occurrence probability of term *t* in a specific GO annotation corpus. Therefore, the Resnik’s similarity between two proteins *P*_*i*_ and *P*_*j*_, annotated to sets of GO terms *G*_*i*_ and *G*_*j*_ respectively, defines the maximum IC of the set *G*_*i*_ × *G*_*j*_ as

6 $$ {Sim}_{Resnik}\left({P}_i,{P}_j\right)=\max \left\{{Sim}_{Resnik}\left({\mathrm{t}}_i,{t}_j\right)|{t}_i\in {G}_i,{t}_j\in {G}_j\right\}. $$

After computing the Resnik’s semantic similarity between any two proteins, we set one of the semantic similarities as the threshold *θ*_*R*_. The *N*_*PPI*_ is then constructed by linking only the proteins with a semantic similarity above *θ*_*R*_. The threshold similarity *θ*_*R*_ is obtained by deriving a reference PPI network, called *N*_*S*_, from the training set of protein pairs. In constructing *N*_*S*_, each protein pair is preclassified as interacting or non-interacting, and two proteins are connected only when confirmed as interacting in the training set. The *θ*_*R*_ is then selected to equalize the average degrees in *N*_*PPI*_ and *N*_*S*_, thereby capturing the PPI characteristics of the training data in *N*_*PPI*_. Based on the topology of *N*_*PPI*_, we create five network-based network features for each protein pair <*P*_*i*_, *P*_*j*_>: (a) number of common neighbors, (b) the Jaccard index, (c) the Adamic–Adar index, (d) the preferential attachment score, and (e) the Otsuka–Ochiai coefficient [43, 44]. The network-based features are formally defined in Table 2. With the similar flexibility of the GO-based features, the network-based features of the same protein pair can be different and adapt when the training data change and so does the topology of the PPI network.

**Table 2:** Summary of network-based features

Features	Definition^a^
Common neighbors	|*N*(*P*_*i*_) ∩ *N*(*P*_*j*_)|
Jaccard index	$\frac{\left|N\left({P}_i\right)\cap N\left({P}_j\right)\right|}{\left|N\left({P}_i\right)\cup N\left({P}_j\right)\right|}$
Adamic–Adar index	$\sum \limits_{P_k\in N\left({P}_i\right)\cap N\left({P}_j\right)}\frac{1}{\log \left|N\left({P}_k\right)\right|}$
Preferential attachment score	|*N*(*P*_*i*_)| × |*N*(*P*_*j*_)|
Otsuka–Ochiai coefficient	$\frac{\left|N\left({P}_i\right)\cap N\left({P}_j\right)\right|}{\sqrt{\left|N\left({P}_i\right)\times N\left({P}_j\right)\right|}}$

^a^*N*(*P*_*i*_) denotes the set of *P*_*i*_’s neighbors

### Stacked generalization

Ensemble learning combines many different classifiers into one predictive unit typically by majority voting. In simple voting schemes such as bagging [45], each classifier is allowed one vote, and the majority vote is accepted as the final prediction. Boosting [46] is a more complex scheme that weights the training examples by the difficulty of classifying them correctly, and updates the rewards to the classifiers based on the weights of their correctly classified examples. The final predictive unit is the weighted average of all classifiers over their rewards.

Unlike the bagging and boosting approaches, which mainly aim to improve the performance of a classifier by reducing the variance of multiple classifiers, our stacked classifiers operate as layered processes that aim to deduce the biases of the base generalizers [24]. In the stacked learning framework, each base classifier in a set is trained on a dataset, and their predictions are assembled as the meta-data. Successive layers of meta-classifiers receive the meta-data as the input for training the meta-models in parallel, then pass their outputs to the subsequent layer. A single classifier at the top level makes the final prediction. Stacked generalization is considered as a form of meta-learning because the transformed training data for the current layer contain the predictive information of the preceding learners, which constitutes a form of meta-knowledge.

We developed a two-level stacked generalization architecture for PPI prediction. The bottom level comprises four base classifiers: RF [47], NB [48], ANN [49] and KNN [50]. At the top level, we place a Radial Basis Function (RBF) kernel SVM [51] as a meta-classifier that arbitrates among the base classifiers, and makes the final prediction. The base classifiers are trained on a set of protein pairs that have been pre-labeled as interacting or non-interacting, and translated to vectors of sequence-based features and GO-based features. The predictions of the base classifiers provide the meta-data for training the top-level SVM. To classify a new protein pair, we first feed its feature vector derived from the physicochemical properties, GO terms, and network topologies to each trained base classifier, which makes a prediction. Subsequently, the predictions of the four classifiers are input to the trained SVM, which makes the final PPI prediction for the new protein pair.

### Datasets

Our PPI-MetaGO for PPI prediction was evaluated on the datasets of eight species: *Homo sapiens*, *Mus musculus*, *Drosophila melanogaster*, *Arabidopsis thaliana*, *Caenorhabditis elegans*, *Saccharomyces cerevisiae*, *Schizosaccharomyces pombe*, and *Escherichia coli*. In the comparative analysis, we used the data collected from different databases and processed in earlier studies, namely, DIP [52], HPRD [53] and MIPS MPact [54]. The species, sizes and prediction tools of the datasets are summarized in Table 3. For species studied by different prediction methods on different datasets, such as *H. sapiens*, Table 4 summarizes the numbers of coincident proteins and protein pairs in the additional datasets. These numbers indicate the degrees of similarity between pairs of datasets, and should consequently be considered when evaluating and comparing the prediction methods.

**Table 3:** Summary of benchmark datasets

Label	Species	Proteins	Interactions (positive/negative)	Prediction Tool
HS1	*Homo sapiens*	9439	37,027/37027	PRED_PPI (Guo et al.)
EC1	*Escherichia coli*	1834	6954/6954	PRED_PPI (Guo et al.)
DM1	*Drosophila melanogaster*	7059	21,975/21975	PRED_PPI (Guo et al.)
CE	*Caenorhabditis elegans*	2640	4030/4030	PRED_PPI (Guo et al.)
SC1	*Saccharomyces cerevisiae*	2245	3956/3956	PRED_PPI (Guo et al.)
HS2^a^	Homo sapiens	7033	24,718/177117	SPRINT (Li & Ilie)
HS3	Homo sapiens	1515	12,244/12244	TRI_tool (Perovic et al.)
SC2	Saccharomyces cerevisiae	3291	15,238/15238	go2ppi-RF (Maetschke et al.)
HS4	Homo sapiens	3296	3490/3490	go2ppi-RF (Maetschke et al.)
EC2	Escherichia coli	589	1167/1167	go2ppi-RF (Maetschke et al.)
SP	Schizosaccharomyces pombe	904	742/742	go2ppi-RF (Maetschke et al.)
AT	*Arabidopsis thaliana*	756	541/541	go2ppi-RF (Maetschke et al.)
MM	*Mus musculus*	1088	500/500	go2ppi-RF (Maetschke et al.)
DM2	Drosophila melanogaster	658	321/321	go2ppi-RF (Maetschke et al.)
SC3	Saccharomyces cerevisiae	2152	3844/3844	go2ppi-RF (Maetschke et al.)
HS5	Homo sapiens	6037	1091/3427	HVSM (Zhang et al.)
SC4	Saccharomyces cerevisiae	5436	4529/10831	HVSM (Zhang et al.)
SC5	Saccharomyces cerevisiae	454	500/500	GIS-MaxEnt (Armean et al.)
SC6	Saccharomyces cerevisiae	4424	17,257/48594	DeepSequencePPI (Gonzalez-Lopez et al.)

^a^In the work of SPRINT [6], the authors prepared three separate data into three human PPI data sets (i.e. C1, C2 and C3). To facilitate 10-fold CV in our experiments, we merged all three data sets into one single set of human PPI data with the redundancies removed

**Table 4:** Summary of different PPI datasets for *Homo sapiens*, *Saccharomyces cerevisiae*, *Escherichia coli*, and *Drosophila melanogaster*. (a) the numbers of coincident proteins and (b) the numbers of coincident interacting and non-interacting protein pairs (Pos and Neg, respectively) in the datasets

(a)						
Protein	HS2	HS3	HS4	HS5		
HS1	4513(11959^a^)	971(9983)	2460 (10275)	2699 (12777)		
HS2	–	1043 (7505)	2272 (8057)	2492 (10578)		
HS3	–	–	620 (4191)	616 (6936)		
HS4	–	–	–	1472 (7861)		
Protein	SC2	SC3	SC4	SC5	SC6	
SC1	1759 (3777)	2078 (2319)	2088 (5593)	0 (2699)	1979 (4690)	
SC2	–	1762 (3681)	3187 (5540)	0 (3745)	2622 (5093)	
SC3	–	–	2074 (5514)	0 (2606)	2001 (4574)	
SC4	–	–	–	0 (5890)	3612 (6248)	
SC5	–	–	–	–	0 (4878)	
Protein	EC2					
EC1	469 (1954)					
Protein	DM2					
DM1	295 (7422)					
(b)						
Pos	HS1	HS2	HS3	HS4	HS5	
Neg						
HS1	–	8388 (53357)	2282 (46989)	1626 (38891)	514 (37604)	
HS2	87 (214057^b^)	–	2742 (34220)	1505 (26703)	451 (25363)	
HS3	5 (49266)	59 (189302)	–	463 (15271)	194 (13141)	
HS4	4 (40513)	15 (180592)	2 (15732)	–	272 (4309)	
HS5	0 (40454)	5 (180539)	1 (15670)	0 (6917)	–	
Pos	SC1	SC2	SC3	SC4	SC5	SC6
Neg						
SC1	–	1985 (17236)	3587 (4213)	3372 (5113)	0 (4456)	3526 (17687)
SC2	4 (19190)	–	2073 (17009)	2534 (17233)	0 (15738)	4479 (28016)
SC3	10 (7790)	8 (19074)	–	3532 (4841)	0 (4344)	3728 (17373)
SC4	4 (14783)	12 (26057)	3 (14672)	–	0 (5029)	3602 (18184)
SC5	0 (4456)	0 (15738)	0 (4344)	0 (11331)	–	0 (17757)
SC6	43 (52507)	76 (63756)	28 (52410)	42 (59383)	0 (49094)	–
Pos	EC1	EC2				
Neg						
EC1	–	384 (7737)				
EC2	3 (8118)	–				
Pos	DM1	DM2				
Neg						
DM1	–	15 (22281)				
DM2	0 (22296)	–				

*HS* Homo sapiens, *SC* Saccharomyces cerevisiae, *EC* Escherichia coli, *DM* Drosophila melanogaster ^a^Numbers in parentheses are the total numbers of non-duplicated proteins in the two datasets, e.g. HS1 and HS2

^b^Numbers in parentheses are the total numbers of non-duplicated protein pairs in the two datasets, e.g. HS1 and HS2

## Results

### Performance measures

To evaluate and compare the performances of PPI-MetaGO and other PPI prediction approaches, we conducted 10-fold cross-validation (CV) using the 7 measures: (1) true positive rate (TPR), (2) false positive rate (FPR), (3) precision, (4) percentage accuracy, (5) F-score, (6) Matthews correlation coefficient (MCC), and (7) area under receiver operating characteristic curve (AUC). The seven performance measures are defined as follows:

7 $$ \mathrm{TPR}=\mathrm{TP}/\left(\mathrm{TP}+\mathrm{FN}\right) $$

8 $$ \mathrm{FPR}=\mathrm{FP}/\left(\mathrm{FP}+\mathrm{TN}\right) $$

9 $$ \mathrm{Precision}=\mathrm{TP}/\left(\mathrm{TP}+\mathrm{FP}\right) $$

10 $$ \mathrm{Accuracy}=\left(\mathrm{TP}+\mathrm{TN}\right)/\left(\mathrm{TP}+\mathrm{TN}+\mathrm{FP}+\mathrm{FN}\right) $$

11 $$ \mathrm{F}-\mathrm{score}=2\times \mathrm{TPR}\times \mathrm{Precision}/\left(\mathrm{TPR}+\mathrm{Precision}\right) $$

12 $$ \mathrm{MCC}=\frac{TP\times TN- FP\times FN}{\sqrt{\left( TP+ FP\right)\left( TP+ FN\right)\left( TN+ FP\right)\left( TN+ FN\right)}} $$

13 $$ \mathrm{AUC}=\mathrm{Area}\ \mathrm{under}\ \mathrm{the}\ \mathrm{ROC}\ \mathrm{curve} $$

where TP, TN, FP, and FN represent true positive, true negative, false positive, and false negative, respectively.

### Performance comparison of PPI-MetaGo and recent PPI predictors

The PPIs predicted by PPI-MetaGO on the different datasets were compared with those of seven recent PPI predictors: PRED_PPI [55], SPRINT [6], TRI_tool [56], hierarchical vector space model (HVSM) [57], go2ppi [19], GIS-MaxEnt [17], and DeepSequencePPI [22]. Among these, PRED_PPI, SPRINT, TRI_tool, and DeepSequencePPI are sequence-based methods, whereas HVSM, go2ppi and GIS-MaxEnt are GO-driven approaches.

Each of these prediction tools was previously trained and tested on a different dataset. In each experiment, we selected one tool for comparison with our proposed approach. To ensure a consistent and unbiased test, we trained and tested PPI-MetaGO exclusively on the training and evaluation datasets of the predictor selected for comparison. The performances of the different PPI prediction methods were evaluated by three times of stratified 10-fold CV. The dataset was randomly divided into 10 disjoint folds (subsets) of approximately equal size. The folds were stratified to maintain the same distribution of the interacting and non-interacting protein pairs as in the original dataset. One fold was retained for testing the prediction performance; the remaining nine folds were used for training. The same training–testing process was iterated on each fold. In each iteration, if the performance of the PPI predictors was sensitive to the parameter values, we optimized all settings in a systematic search (sequential or grid search) within a range of parameter values, and used the values yielding the best prediction. The result of each test run on the selected fold was pooled. After completing all iterations of the 10-fold CV, the results of all runs were averaged to obtain the overall performance of the predictor. The results are shown in Table 5. The ACC, F-score, MCC and AUC performances of PPI-MetaGO and the other PPI predictors were compared in paired *t*-tests. Conventionally, significant differences in comparison tables are marked with an asterisk. However, the asterisks in Table 5 indicate *insignificant* differences, highlighting that in most cases, PPI-MetaGO significantly outperforms the established prediction tool. Note that in Table 5 some of the values of AUC are higher than those of ACC, F-score, and MCC for the same dataset, such as in HS1 and SC2. This is because AUC is defined as the area under the ROC curve, which depicts the tradeoffs between true positives and false positives, while any of the other performance measures (e.g. ACC) merely corresponds to a single point in the ROC space, depending on the output score threshold specified for the prediction tools. To warrant the best performance of each tool for the CVs, we chose the threshold value that maximized the MCC in the training phase, and used that threshold for predicting PPI in the test phase of the CVs.

**Table 5:** Performance results of 10-fold CV of PPI prediction methods

	PPI-MetaGO							Other recent prediction tools							
Dataset	TPR	FPR	Prec	ACC	F-score	MCC	AUC	TPR	FPR	Prec	ACC	F-score	MCC	AUC	Tool
HS1	0.964	0.013	0.987	0.975	0.975	0.951	0.993	0.835	0.046	0.948	0.895	0.888	0.795	0.900	PRED_PPI
EC1	0.923	0.015	0.984	0.954	0.952	0.909	0.983	0.897	0.147	0.860	0.875	0.878	0.752	0.935	PRED_PPI
DM1	0.966	0.010	0.990	0.978	0.978	0.956	0.996	0.750	0.223	0.771	0.763	0.760	0.527	0.841	PRED_PPI
CE	0.984	0.004	0.995	0.990	0.990	0.979	0.997	0.833	0.158	0.841	0.838	0.837	0.676	0.910	PRED_PPI
SC1	0.898	0.051	0.947	0.923	0.921	0.848	0.974	0.686	0.342	0.667	0.672	0.676	0.344	0.737	PRED_PPI
HS2	0.327	0.009	0.834	0.91	0.469	0.487	0.791	0.540	0.072	0.513	0.881	0.526	0.458	0.814	SPRINT
HS3	0.826	0.187	0.816	0.820	0.821	0.639	0.897	0.789	0.193	0.803	0.798	0.796	0.596	0.878	TRI_tool
SC2	0.858	0.059	0.936	0.899	0.895	0.802	0.952	0.819	0.076	0.915	0.872	0.864	0.747	0.921	go2ppi-RF
HS4	0.826	0.106	0.887	0.860	0.855	0.723	0.921	0.786	0.126	0.863	0.830	0.822	0.663	0.890	go2ppi-RF
EC2	0.879	0.075	0.922	0.902^*^	0.900^*^	0.805^*^	0.950^*^	0.869	0.059	0.937	0.905	0.902	0.813	0.951	go2ppi-RF
SP	0.922	0.065	0.935	0.929	0.928	0.858	0.965	0.865	0.096	0.901	0.885	0.882	0.771	0.941	go2ppi-RF
AT	0.778	0.163	0.830	0.808^*^	0.801	0.619^*^	0.866	0.684	0.105	0.875	0.789	0.764	0.596	0.810	go2ppi-RF
MM	0.754	0.182	0.808	0.786	0.779	0.575	0.860	0.604	0.128	0.836	0.738	0.695	0.500	0.762	go2ppi-RF
DM2	0.857	0.118	0.885	0.869	0.867	0.744	0.916	0.832	0.146	0.853	0.843	0.841	0.688	0.889	go2ppi-RF
SC3	0.786	0.104	0.883	0.841	0.831	0.686	0.894	0.707	0.120	0.858	0.794	0.774	0.598	0.826	go2ppi-RF
HS5	0.824	0.026	0.911	0.938	0.864	0.826	0.974	0.782	0.213	0.801	0.784	0.609	0.578	0.849	HVSM
SC4	0.773	0.036	0.901	0.908	0.832	0.773	0.945	0.707	0.213	0.777	0.747	0.581	0.505	0.797	HVSM
SC5	0.920	0.034	0.965	0.943	0.942	0.888	0.984	0.926	0.088	0.915	0.919	0.920	0.839	0.977	GIS-MaxEnt
SC6	0.912	0.064	0.934	0.924	0.923	0.848	0.972	0.920	0.078	0.942	0.932	0.931	0.864	0.978	DeepSequencePPI

*TPR* true positive rate, *FPR* false positive rate, *Prec* precision, *ACC* accuracy, *MCC* Matthews correlation coefficient, *AUC* area under ROC

*HS* Homo sapiens, *EC* Escherichia coli, *DM* Drosophila melanogaster, *CE* Caenorhabditis elegans, *SC Saccharomyces cerevisiae, SP schizosaccharomyces* pombe, *AT* Arabidopsis thaliana, *MM* Mus musculus

^*^denotes *insignificant* difference in a paired *t*-test between PPI-MetaGO and the prediction tool in the 10-fold CV at the significance level *α* = 0.05

Based on SVM, PRED_PPI [55] was developed for predicting PPIs in humans, yeast, Drosophila, *E. coli*, and *C. elegans*. As shown in Table 5, the ACC, F-score, MCC, and AUC of the HS1, EC1, DM1, CE, and SC1 datasets (on which PRED_PPI was trained and tested) were significantly higher in PPI-MetaGO than in PRED_PPI (paired *t*-test, *p* < 0.05). The superiority of PPI-MetaGO could be attributable to the inclusion of GO-based and network-based features in its protein-pair representation, and the synergy of multiple base classifiers in its learning. Unlike PRED_PPI, both SPRINT [6] and TRI_tool [56] were specifically developed for PPI predictions in humans. SPRINT was designed for predicting the entire human interactome, whereas TRI_tool is a web-based online tool that automatically predicts transcriptional regulation interactions in humans. We compared PPI-MetaGO with SPRINT on the human PPI dataset HS2 (on which SPRINT was trained and tested). SPRINT applies an alignment algorithm that evaluates the contributions of similar protein subsequences to the likelihood of protein interactions. In contrast, the sequence-based features in PPI-MetaGO were derived from the physicochemical properties of amino acids. Although the ACC and MCC were significantly higher in PPI-MetaGO than in SPRINT (paired *t*-test, *p* < 0.05), the F-score and AUC were lower than in SPRINT, probably because SPRINT is designed specifically for human PPI prediction, and has been carefully trained on human PPI data. The final sequence-based PPI predictor competed against PPI-MetaGO was a web-tool called TRI_tool, which predicts PPIs using a pseudo amino-acid composition representation and an RF classifier. In this comparison, PPI-MetaGO and TRI_tool were tested on HS3 (on which TRI_tool was trained and evaluated). The ACC, F-score, MCC, and AUC were significantly higher in PPI-MetaGO than in TRI_tool (paired *t-*test, *p* < 0.05) although the improvement in PPI-MetaGo was modest. Instead of relying on hand-crafted features to represent a protein pair for PPI prediction in deep learning [20, 21], DeepSequencePPI [22] learns low-level features directly from raw protein sequences by combining the embedding techniques with recurrent neural networks. We compared PPI-MetaGO and DeepSequencePPI on SC6, on which DeepSequencePPI had been earlier trained and tested. Compared with the other datasets collected from *Saccharomyces cerevisiae*, SC6 has the largest size in terms of the number of interacting and non-interacting protein pairs, respectively. The dataset size has a greater impact on deep learners than on other predictors because deep learning engages in feature extraction from raw data before constructing the prediction model. As a result, DeepSequencePPI could have more leverage with large datasets, such as SC6, than PPI-MetaGO. While the ACC, F-score, MCC, and AUC were significantly higher in DeepSequencePPI than in PPI-MetaGO (paired *t*-test, *p* < 0.05), the differences were marginal.

In addition to the sequence-based methods, we selected three state-of-the-art GO-driven approaches for comparison with PPI-MetaGO. To facilitate the paired comparisons with PPI-MetaGO, we tested each GO-driven approach on all three categories of GO terms, rather than sequentially evaluating the performance on each category. As a hybrid approach go2ppi [19] combines semantic similarity measures (SSMs) and ML. PPI-MetaGO and go2ppi-RF (using Random Forest) were evaluated on the eight datasets previously used for training and testing go2ppi. In six out of the eight datasets, except EC2 and AT, PPI-MetaGO significantly outperformed go2ppi-RF for all four measures, ACC, F-score, MCC and AUC (paired *t-*test, *p* < 0.05). PPI-MetaGO performed significantly better than go2ppi-RF for all measures except AUC in the EC2 dataset, and the differences in ACC and MCC were insignificant in the AT dataset (as indicated by the asterisks in Table 5). Rather than adopting a hybrid approach, HVSM refines the basic vector space model (VSM) approaches by relating the terms in the hierarchical structure of GO DAG. The method considers not only the directly annotated GO terms, but also their ancestors and descendants. The HVSM improves the expressiveness of the gene vectors transformed from GO terms, which should improve the accuracy of the similarity measure between vector pairs. We compared PPI-MetaGO and HVSM on HS5 and SC4, on which HVSM had been earlier trained and tested. In an evaluation study, the similarity measure of HSVM achieved a higher AUC on HS5 and SC4 [57] than several popular SSMs, including TCSS [13] and Resnik’s measure [40]. However, PPI-MetaGO, which adopts Resnik’s measure in its hybrid approach, outperformed HVSM in AUC and all other performance measures (see Table 5). The third annotation-based method selected for a performance comparison with PPI-metaGO was GIS-MaxEnt. Unlike go2ppi and HVSM, GIS-MaxEnt incorporates two annotation sources, GO and InterPro, and processes them by a maximum entropy modeling method, thus preparing an input matrix for training the SVM in PPI prediction. We compared the performances of PPI-MetaGO and GIS-MaxEnt on the SC5 dataset, on which GIS-MaxEnt had been previously evaluated. PPI-MetaGO significantly outperformed GIS-MaxEnt for all four performance measures (ACC, F-score, MCC and AUC; paired *t-*test, *p* < 0.05).

### Study of cross-species PPI predictions

In addition to intra-species self-tests, cross-species PPI prediction has been reported in several previous studies [19, 58]. In these studies, the PPI predictor was trained on one species, and then tested on others. According to Park’s [58] results, the cross-species predictive performances of sequence-based PPI predictors are considerably lower than intra-species self-test performances. An AUC of 0.9 achieved by 4-fold CV on a human dataset can decrease to 0.68 if the predictor was trained from yeast before application to the human dataset. In contrast to sequence-based prediction methods, Maetschke et al. [19] hypothesized that GO-based predictors can maintain good cross-species predictive performances because GO was designed as a species-independent annotation system. They separately tested go2ppi with an NB classifier on seven species in the BP, CC, and MF ontologies, and concluded that good prediction performance in the cross-species prediction requires a high intra-species self-test performance. That is, the predictive performance on the target species was high when the self-test performance for that species was also high; otherwise, the cross-species performance was low.

Following Maetschke et al. [19], we conducted the cross-species 10-fold CV tests of PPI-MetaGO and go2ppi-NB on the same datasets of the same seven species, using the BP, CC and MF ontologies separately. The AUCs are summarized in Table 6. The intra-species self-test results are shown diagonally in the cells in boldface for reference.

**Table 6:** AUCs of cross-species predictions of PPI-MetaGO/go2ppi-NB using the biological process (BP), cellular component (CC), and molecular function (MF) ontology, respectively

	BP	*Test*						
Train	AUC	*EC2*	*SP*	*HS4*	*SC2*	*DM2*	*AT*	*MM*
	EC2	0.94/0.88	0.92/0.78	0.86/0.76	0.87/0.77	0.69/0.80	0.73/0.64	0.59/0.65
	SP	0.87/0.65	0.96/0.81	0.88/0.74	0.87/0.75	0.68/0.74	0.78/0.55	0.60/0.61
	HS4	0.90/0.72	0.94/0.75	0.95/0.76	0.88/0.73	0.71/0.80	0.76/0.64	0.63/0.68
	SC2	0.89/0.80	0.95/0.79	0.90/0.76	0.95/0.79	0.76/0.83	0.73/0.67	0.58/0.70
	DM2	0.83/0.60	0.92/0.70	0.85/0.71	0.87/0.67	0.79/0.78	0.68/0.63	0.58/0.60
	AT	0.82/0.72	0.91/0.80	0.84/0.74	0.86/0.75	0.73/0.76	0.86/0.72	0.61/0.63
	MM	0.81/0.62	0.87/0.71	0.85/0.73	0.85/0.71	0.70/0.74	0.69/0.56	0.73/0.69
	CC	*Test*						
Train	AUC	*EC2*	*SP*	*HS4*	*SC2*	*DM2*	*AT*	*MM*
	EC2	0.94/0.88	0.91/0.67	0.86/0.68	0.87/0.68	0.68/0.74	0.71/0.59	0.55/0.66
	SP	0.85/0.55	0.96/0.82	0.88/0.70	0.88/0.78	0.66/0.73	0.73/0.61	0.56/0.56
	HS4	0.89/0.70	0.94/0.70	0.95/0.80	0.88/0.77	0.71/0.79	0.75/0.65	0.66/0.68
	SC2	0.89/0.78	0.95/0.74	0.90/0.76	0.94/0.83	0.75/0.80	0.72/0.65	0.58/0.64
	DM2	0.82/0.64	0.91/0.69	0.84/0.74	0.87/0.79	0.81/0.80	0.70/0.63	0.58/0.60
	AT	0.79/0.57	0.90/0.66	0.84/0.67	0.85/0.73	0.67/0.70	0.85/0.71	0.61/0.61
	MM	0.76/0.70	0.87/0.71	0.86/0.74	0.85/0.77	0.68/0.77	0.61/0.61	0.70/0.70
	MF	*Test*						
Train	AUC	*EC2*	*SP*	*HS4*	*SC2*	*DM2*	*AT*	*MM*
	EC2	0.94/0.88	0.92/0.65	0.87/0.62	0.87/0.66	0.69/0.70	0.74/0.62	0.57/0.56
	SP	0.85/0.81	0.97/0.76	0.87/0.65	0.87/0.67	0.68/0.72	0.76/0.67	0.58/0.57
	HS4	0.89/0.85	0.94/0.78	0.95/0.76	0.88/0.68	0.72/0.76	0.75/0.67	0.63/0.68
	SC2	0.88/0.89	0.95/0.73	0.89/0.66	0.95/0.76	0.75/0.75	0.74/0.59	0.55/0.62
	DM2	0.79/0.80	0.92/0.68	0.85/0.65	0.86/0.66	0.82/0.79	0.72/0.67	0.57/0.60
	AT	0.75/0.72	0.93/0.70	0.83/0.63	0.83/0.60	0.72/0.70	0.86/0.75	0.61/0.58
	MM	0.79/0.77	0.88/0.66	0.86/0.67	0.85/0.65	0.67/0.74	0.71/0.70	0.72/0.67

*EC Escherichia coli, SP schizosaccharomyces* pombe, *HS* Homo sapiens, *SC* Saccharomyces cerevisiae, *DM* Drosophila melanogaster, *AT* Arabidopsis thaliana, *MM* Mus musculus

From Table 6, we note that PPI-MetaGO and go2ppi-NB achieved (almost) maximum AUCs on all self-tests, and the AUCs were usually higher than obtained from cross-species tests. Compared with PPI-MetaGO, go2ppi-NB produced substantially lower self-test and cross-species AUCs in most cases. Consistent with previous studies, the performance on the target species was high (low) when the self-test performance on that species was also high (low). In both Maetschke et al.’s and our study, the AUCs of the self-tests and cross-species tests were lowest on the mouse PPI dataset (the MM dataset; see final column of Table 6). Notably however, when PPI-MetaGO was trained on MM, it achieved reasonably high AUCs tested on the datasets of other species.

## Discussion

We introduced an ensemble meta learning approach, PPI-MetaGO, for PPI prediction that integrated different protein-pair representations. To demonstrate its performances, we compared PPI-MetaGO with seven other PPI prediction tools on 19 protein datasets from eight species. Based on the design of PPI-MetaGO and the results of the experiments, we identified three issues worth further discussion. First, while Table 5 shows the superiority of PPI-MetaGO using a combination of three types of features, could other feature combinations produce the same level of synergy, and to what degree did they affect the prediction performances? Second, different benchmark datasets, even collected from the same species (e.g. HS1~HS5), have been used in previous studies of PPI prediction (see Table 3). How significant was data selection for evaluating the performances? Third, the GO-based and network-based features employed in PPI-MetaGO are obtained based on the partitioning of a GO term hierarchy and the topological properties of a PPI network, respectively. As the training data determine both the GO hierarchy clustering and the PPI network, the GO-based and network-based features can both vary when different training data are provided. How did they accommodate to the change of the benchmark datasets for the same species such as *H. sapiens*? We discuss these issues as follows.

### Synergy of different feature combinations

PPI-MetaGO constructs a meta-classification model for PPI prediction using three types of features: physicochemical features, LCA-indexed GO-term features, and network-based features. For simplicity, we denote the feature types by F_1_, F_2_, and F_3_, respectively. The effects of combining F_1_, F_2_, and F_3_ were summarized in Table 7, but a comparison with other feature combinations can provide insight into the importance of different feature types in PPI prediction. For this purpose, we tested all possible feature combinations in PPI-MetaGO on the same datasets, and analyzed their effects on the prediction performance. The results of different feature combinations on some PPI datasets are given in Table 7. As shown by the synergy of the F_1_, F_2_ and F_3_ features in Table 7, the PPI prediction was superior on most datasets, but some combinations or even single feature types maximized the performance on certain data sets. On the HS1, HS3, and SC2 datasets, the performance of PPI-MetaGo was generally higher for the combined three feature types than for the other feature configurations. However, on the DM1 dataset, the highest ACC, F_1_ score, and MCC were obtained in PPI-MetaGo with the F_1_ features alone. Meanwhile, the best achievement on EC2 was obtained by PPI-MetaGo with the F_1_ and F_2_ features. The performance discrepancies after varying the feature combinations suggest that each feature type makes a distinct contribution to the PPI prediction on different datasets.

**Table 7:** Performance results in 10-fold CV of PPI-MetaGO with different feature combinations

	TPR	FPR	Prec	ACC	F-score	MCC	AUC
HS1							
F_1_	0.917	0.016	0.983	0.951	0.949	0.903	0.981
F_2_	0.872	0.101	0.897	0.886	0.884	0.772	0.901
F_3_	0.686	0.637	0.521	0.525	0.588	0.053	0.534
F_1_&F_2_	0.894	0.031	0.966	0.931	0.929	0.865	0.977
F_1_&F_3_	0.926	0.028	0.971	0.949	0.948	0.899	0.987
F_2_&F_3_	0.885	0.089	0.909	0.898	0.896	0.796	0.915
F_1_&F_2_&F_3_	0.964	0.013	0.987	0.975	0.975	0.951	0.993
DM1							
F_1_	0.978	0.001	0.999	0.988	0.988	0.977	0.997
F_2_	0.664	0.237	0.727	0.714	0.644	0.449	0.787
F_3_	0.690	0.604	0.417	0.543	0.497	0.132	0.538
F_1_&F_2_	0.933	0.005	0.995	0.964	0.963	0.930	0.995
F_1_&F_3_	0.977	0.001	0.999	0.988	0.988	0.976	0.998
F_2_&F_3_	0.740	0.253	0.728	0.743	0.705	0.501	0.765
F_1_&F_2_&F_3_	0.966	0.010	0.990	0.978	0.978	0.956	0.996
HS3							
F_1_	0.812	0.215	0.790	0.798	0.801	0.597	0.862
F_2_	0.730	0.244	0.750	0.743	0.740	0.487	0.788
F_3_	0.626	0.235	0.731	0.695	0.672	0.397	0.733
F_1_&F_2_	0.809	0.206	0.797	0.801	0.803	0.602	0.870
F_1_&F_3_	0.812	0.191	0.809	0.811	0.811	0.621	0.883
F_2_&F_3_	0.720	0.202	0.781	0.759	0.749	0.520	0.810
F_1_&F_2_&F_3_	0.826	0.187	0.816	0.820	0.821	0.639	0.897
SC2							
F_1_	0.747	0.261	0.741	0.743	0.744	0.486	0.812
F_2_	0.805	0.133	0.858	0.836	0.831	0.673	0.871
F_3_	0.796	0.063	0.927	0.866	0.856	0.740	0.885
F_1_&F_2_	0.809	0.145	0.848	0.832	0.828	0.665	0.908
F_1_&F_3_	0.841	0.070	0.923	0.885	0.880	0.774	0.933
F_2_&F_3_	0.858	0.065	0.930	0.897	0.893	0.796	0.934
F_1_&F_2_&F_3_	0.858	0.059	0.936	0.899	0.895	0.802	0.952
EC2							
F_1_	0.763	0.169	0.821	0.797	0.790	0.598	0.845
F_2_	0.810	0.089	0.902	0.860	0.853	0.725	0.913
F_3_	0.878	0.075	0.922	0.902	0.899	0.805	0.938
F_1_&F_2_	0.793	0.141	0.850	0.826	0.820	0.655	0.895
F_1_&F_3_	0.901	0.069	0.929	0.916	0.914	0.832	0.956
F_2_&F_3_	0.915	0.046	0.952	0.934	0.933	0.870	0.973
F_1_&F_2_&F_3_	0.913	0.048	0.950	0.933	0.931	0.866	0.960

*F*_*1*_ physicochemical features, *F*_*2*_ LCA-indexed GO-term features, *F*_*3*_ network-based features

*TPR* true positive rate, *FPR* false positive rate, *Prec* precision, *ACC* accuracy, *MCC* Matthews correlation coefficient, *AUC* area under ROC

*EC* Escherichia coli, *HS* Homo sapiens, *SC* Saccharomyces cerevisiae, *DM* Drosophila melanogaster

### Effects of training data on prediction performances

PPI-MetaGO generally outperformed its competing tools, as shown in Table 5, while we also observed that its performances could vary among different datasets even from the same species. For example, for *H. sapiens* it performed the best on HS1 for AUC as high as 0.993, but did poorly on HS2 with a markedly lower 0.791 AUC. According to Table 4, the contents of the datasets from the same species, HS1 to HS5 for example, can differ significantly as indicated by the small numbers, relative to the dataset sizes, of the proteins and protein pairs commonly shared between any pair of datasets. In addition, the non-interacting protein pairs, namely, negative examples, common to two datasets, such as HS2 and HS3, are markedly limited. The non-interacting protein pairs in HS4 and HS5 are entirely different, as shown in Table 4(b). These differences in the datasets can affect the training of any predictor, and consequently its predictive performance, as noted from Table 5. To evaluate the effects of different negative examples on the prediction performances, we trained and tested PPI-MetaGO and other PPI predictors based on mixed positive and negative data from different datasets. We conducted a 10-fold CV test of PPI-MetaGO and HVSM, using the original positive examples of HS5, but replacing its original negative examples with those from HS4. We also compared PPI-MetaGO with DeepSequencePPI, using only the positive examples of SC6, but combined with the negative examples of SC2. The results are shown in Table 8. Compared with the results of HS5 in Table 5, we noted that the performances of HVSM increased substantially while PPI-MetaGO’s performances decreased by a narrow margin. Despite the opposite effects of the new negative data on HVSM and PPI-MetaGO, respectively, PPI-MetaGO still outperformed HVSM on the new dataset. By contrast, after the replacement of the negative examples in SC6, the performances of both DeepSequencePPI and PPI-MetaGO declined markedly. Notably however, DeepSequencePPI’s performances decreased by a wider margin than PPI-MetaGO’s, which made PPI-MetaGO superior on this new dataset. These results suggest the importance of the selection of data for training and evaluating PPI predictors.

**Table 8:** Performance results of 10-fold CV, using mixed positive and negative data from different datasets

	PPI-MetaGO							Other recent prediction tools							
Dataset	TPR	FPR	Prec	ACC	F-score	MCC	AUC	TPR	FPR	Prec	ACC	F-score	MCC	AUC	Tool
HS5(+)^a^ HS4(−)	0.811	0.031	0.893	0.932	0.850	0.808	0.971	0.730	0.054	0.808	0.894	0.766	0.700	0.932	HVSM
SC6(+)^b^ SC2(−)	0.810	0.155	0.858	0.826	0.832	0.656	0.901	0.819	0.204	0.824	0.811	0.822	0.621	0.891	DeepSequencePPI

*TPR* true positive rate, *FPR* false positive rate, *Prec* precision, *ACC* accuracy, *MCC* Matthews correlation coefficient, *AUC* area under ROC

*HS* Homo sapiens, *SC* Saccharomyces cerevisiae

^a^Combination of positive data from HS5 and negative data from HS4

^b^Combination of positive data from SC6 and negative data from SC2

### Adaptive generation of features

Unlike most current GO-term features that are node-based, edge-based or hybrid, the proposed GO-based F_2_ features are instead derived from the partitioning of the GO DAG. Other GO-based features are mostly constant-valued; that is, their values for any protein pair remain constant after being determined, and any change of the protein pair dataset for training does not affect the values. By contrast, the proposed F_2_ features of a protein pair are able to adapt to the changes of the training data because the partitioning of the GO DAG depends on the given training set of protein pairs (see Methods). Table 9 shows the numbers of F_2_ features derived from different GO categories in each run of a 10-fold CV for HS1. As indicated in Table 9, the numbers of the generated F_2_ features varied according to different training data, and consequently their values for a protein pair were also adjusted to accommodate to the change. In addition, Table 10 shows the average numbers of the F_2_ features generated for HS1 to HS5 of *H. sapiens*. The high variance of the numbers of F_2_ features generated for the different datasets from the same species suggests the high adaptability of the F_2_ features. By contrast, the values of other GO-based features after being determined to describe a protein pair will remain the same regardless of the datasets. This flexible property enables the F_2_ features to better adapt to new training data when available to improve predictive performances.

**Table 9:** Numbers of F_2_ features generated in each run of 10-fold CV on HS1

Run	Number of F_2_ (ontology CC)	Number of F_2_ (ontology BP)	Number of F_2_ (ontology MF)	Total F_2_
1	83	327	329	739
2	84	324	328	736
3	86	327	331	744
4	83	326	335	744
5	84	325	334	743
6	85	328	333	746
7	82	323	328	733
8	82	327	331	740
9	86	318	330	734
10	82	326	328	736

*F*_*2*_ LCA-indexed GO-term features

**Table 10:** Average numbers of F_2_ features generated for HS1 to HS5 of *H. sapiens*

Dataset	Avg Number of F_2_ (ontology CC)	Avg Number of F_2_ (ontology BP)	Avg Number of F_2_ (ontology MF)	Avg Total F_2_
HS1	84	325	331	740
HS2	103	448	469	1020
HS3	28	29	54	111
HS4	31	78	118	227
HS5	33	105	134	272

*F*_*2*_ LCA-indexed GO-term features, *HS* Homo sapiens

Similar to the F_2_ features, the proposed network-based F_3_ features can also accommodate to the changes in the training data. The F_3_ features are based on the topology of a PPI network constructed from the training data. The change in the training data may consequently alter the topology of the PPI network, and affect the F_3_ features. In contrast to F_2_, the adaptability of F_3_ does not modify the number of features while it revises the feature values for accommodating to the change in the training data. It is computationally prohibited to evaluate every change in the values of F_3_ features due to the change of the training data in the experiments. Nevertheless, the combination of F_3_ with F_1_, F_2_, or both generally produced higher predictive performances than F_1_ or F_2_ alone, as shown in Table 7. These findings fairly verify the contribution of F_3_.

## Conclusions

Researchers have proposed various computational methods for predicting PPIs. These methods are characterized by two primary aspects: (a) the computational strategy that classifies the protein interactions, such as semantic similarity comparisons versus supervised ML approaches, and (b) the representation describing the protein pairs, such as amino acid properties versus GO annotations. These differences in the design philosophies affect the prediction performances of the methods. This study presented an ensemble meta-learning approach for PPI prediction, which utilizes the synergy of multiple ML algorithms and different protein-pair representations to improve the PPI prediction.

The performance of our proposed method, called PPI-MetaGO, was extensively compared with those of seven competitive PPI predictors on 19 protein datasets covering eight species. The experimental results demonstrated the favorable performances of PPI-MetaGO over other PPI predictors. The AUC of PP-MetaGo exceeded 0.9 on 14 out of the 19 datasets, reaching 0.95 or higher in 11 datasets. Following previous works, we also ran cross-species PPI prediction tests. Again, the AUCs of PP-MetaGo were generally high, exceeding those of the competitive predictors in most of the cross-species PPI prediction tests. Overall, these results verify the feasibility and superiority of the proposed ensemble meta-learning approach in PPI prediction. Moreover, as a wider variety of ML algorithms becomes available for base learning, more ontologies emerge for improving the annotations of biological entities or experimental assays, and the flexibility increases for building a stacked architecture appropriate to a certain prediction task, the proposed ensemble meta-learning strategy should become extendible to other domains.

## Data Availability

The datasets supporting the conclusions of this article are available in the Github repository, https://github.com/mlbioinfolab/ppi-metago.

## Abbreviations

10-fold CV: 10-fold cross-validation
AC: Auto covariance
ANN: Artificial neural network
AUC: Area under receiver operating characteristic curve
BP: Biological process
CC: Cellular component
DAG: Directed acyclic graph
FP: False positive
FPR: False positive rate
GO: Gene ontology
HVSM: Hierarchical vector space model
KNN: K-nearest-neighbor
LCA: Lowest common ancestor
MCC: Matthews correlation coefficient
MF: Molecular function
ML: Machine learning
NB: Naïve Bayesian
RF: Random forest
SSMs: Semantic similarity measures
SVM: Support vector machine
TP: True positive
TPR: True positive rate
ULCA: Up to the lowest common ancestor
VSM: Vector space model

## References

[1] Alberts B (1998). The cell as a collection of protein machines: preparing the next generation of molecular biologists. Cell.

[2] Ito T, Chiba T, Ozawa R, Yoshida M, Hattori M, Sakaki Y (2001). A comprehensive two-hybrid analysis to explore the yeast protein interactome. Proc Natl Acad Sci U S A.

[3] Ho Y, Gruhler A, Heilbut A, Bader GD, Moore L, Adams SL, Millar A, Taylor P, Bennett K, Boutilier K (2002). Systematic identification of protein complexes in Saccharomyces cerevisiae by mass spectrometry. Nature.

[4] Zhu H, Bilgin M, Bangham R, Hall D, Casamayor A, Bertone P, Lan N, Jansen R, Bidlingmaier S, Houfek T (2001). Global analysis of protein activities using proteome chips. Science.

[5] von Mering C, Krause R, Snel B, Cornell M, Oliver SG, Fields S, Bork P (2002). Comparative assessment of large-scale data sets of protein-protein interactions. Nature.

[6] Li YW, Ilie L (2017). SPRINT: ultrafast protein-protein interaction prediction of the entire human interactome. BMC Bioinformatics.

[7] Huang YA, You ZH, Chen X, Chan K, Luo X (2016). Sequence-based prediction of protein-protein interactions using weighted sparse representation model combined with global encoding. BMC Bioinformatics.

[8] Guo YZ, Yu LZ, Wen ZN, Li ML (2008). Using support vector machine combined with auto covariance to predict proteinprotein interactions from protein sequences. Nucleic Acids Res.

[9] Tuncbag N, Gursoy A, Nussinov R, Keskin O (2011). Predicting protein-protein interactions on a proteome scale by matching evolutionary and structural similarities at interfaces using PRISM. Nat Protoc.

[10] Zhang LV, Wong SL, King OD, Roth FP (2004). Predicting co-complexed protein pairs using genomic and proteomic data integration. BMC Bioinformatics.

[11] Pesquita C, Faria D, Falcao AO, Lord P, Couto FM (2009). Semantic similarity in biomedical ontologies. PLoS Comput Biol.

[12] Guo X, Liu RX, Shriver CD, Hu H, Liebman MN (2006). Assessing semantic similarity measures for the characterization of human regulatory pathways. Bioinformatics.

[13] Jain S, Bader GD (2010). An improved method for scoring protein-protein interactions using semantic similarity within the gene ontology. BMC Bioinformatics.

[14] Wu XM, Zhu L, Guo J, Zhang DY, Lin K (2006). Prediction of yeast protein-protein interaction network: insights from the gene ontology and annotations. Nucleic Acids Res.

[15] Ben-Hur A, Noble WS (2005). Kernel methods for predicting protein-protein interactions. Bioinformatics.

[16] Bandyopadhyay S, Mallick K (2017). A new feature vector based on gene ontology terms for protein-protein interaction prediction. IEEE-ACM Trans Comput Biol Bioinform.

[17] Armean IM, Lilley KS, Trotter MB, Pilkington NCV, Holden SB (2018). Co-complex protein membership evaluation using maximum entropy on GO ontology and InterPro annotation. Bioinformatics.

[18] Patil A, Nakamura H (2005). Filtering high-throughput protein-protein interaction data using a combination of genomic features. BMC Bioinformatics.

[19] Maetschke SR, Simonsen M, Davis MJ, Ragan MA (2012). Gene ontology-driven inference of protein-protein interactions using inducers. Bioinformatics.

[20] Sun TL, Zhou B, Lai LH, Pei JF (2017). Sequence-based prediction of protein protein interaction using a deep-learning algorithm. BMC Bioinformatics.

[21] Du X, Sun S, Hu C, Yao Y, Yan Y, Zhang Y (2017). DeepPPI: boosting prediction of protein–protein interactions with deep neural networks. J Chem Inf Model.

[22] Gonzalez-Lopez F, Morales-Cordovilla JA, Villegas-Morcillo A, Gomez AM, Sanchez V. End-to-end prediction of protein-protein interaction based on embedding and recurrent neural networks. IEEE Intl Conf Bioinform Biomed (BIBM). 2018:2344–50.

[23] Mitchell TM (1997). Machine learning. AI Mag.

[24] Wolpert DH (1992). Stacked generalization. Neural Netw.

[25] Emini EA, Hughes JV, Perlow DS, Boger J (1985). Induction of hepatitis-a virus-neutralizing antibody by a virus-specific synthetic peptide. J Virol.

[26] Janin J, Wodak S, Levitt M, Maigret B (1978). Conformation of amino-acid side-chains in proteins. J Mol Biol.

[27] Karplus PA, Schulz GE (1985). Prediction of chain flexibility in proteins - a tool for the selection of peptide antigens. Naturwissenschaften.

[28] Kolaskar AS, Tongaonkar PC (1990). A Semiempirical method for prediction of antigenic determinants on protein antigens. FEBS Lett.

[29] Parker JMR, Guo D, Hodges RS (1986). New hydrophilicity scale derived from high-performance liquid-chromatography peptide retention data - correlation of predicted surface residues with antigenicity and X-ray-derived accessible sites. Biochemistry-Us.

[30] Pellequer JL, Westhof E, Vanregenmortel MHV (1993). Correlation between the location of antigenic sites and the prediction of turns in proteins. Immunol Lett.

[31] Ponnuswamy PK, Prabhakaran M, Manavalan P (1980). Hydrophobic packing and spatial arrangement of amino-acid-residues in globular-proteins. Biochim Biophys Acta.

[32] You ZH, Lei YK, Zhu L, Xia JF, Wang B. Prediction of protein-protein interactions from amino acid sequences with ensemble extreme learning machines and principal component analysis. BMC Bioinformatics. 2013;14(Suppl 8):S10. 10.1186/1471-2105-14-S8-S10 PMC3654889 23815620

[33] Saha S, Raghava GPS (2004). BcePred: prediction of continuous B-cell epitopes in antigenic sequences using physico-chemical properties. Lect Notes Comput Sci.

[34] Wold S, Jonsson J, Sjostrom M, Sandberg M, Rannar S (1993). DNA and peptide sequences and chemical processes Multivariately modeled by principal component analysis and partial least-squares projections to latent structures. Anal Chim Acta.

[35] Ashburner M, Ball CA, Blake JA, Botstein D, Butler H, Cherry JM, Davis AP, Dolinski K, Dwight SS, Eppig JT (2000). Gene ontology: tool for the unification of biology. Nat Genet.

[36] Jensen LJ, Gupta R, Staerfeldt HH, Brunak S (2003). Prediction of human protein function according to gene ontology categories. Bioinformatics.

[37] Schlicker A, Domingues FS, Rahnenfuhrer J, Lengauer T (2006). A new measure for functional similarity of gene products based on gene ontology. BMC Bioinformatics.

[38] Wu HW, Su ZC, Mao FL, Olman V, Xu Y (2005). Prediction of functional modules based on comparative genome analysis and gene ontology application. Nucleic Acids Res.

[39] Lord PW, Stevens RD, Brass A, Goble CA (2003). Investigating semantic similarity measures across the gene ontology: the relationship between sequence and annotation. Bioinformatics.

[40] Resnik P (1995). Using information content to evaluate semantic similarity in a taxonomy. *arXiv preprint cmp-lg/9511007*.

[41] Wang JZ, Du ZD, Payattakool R, Yu PS, Chen CF (2007). A new method to measure the semantic similarity of GO terms. Bioinformatics.

[42] Mistry M, Pavlidis P (2008). Gene ontology term overlap as a measure of gene functional similarity. BMC Bioinformatics.

[43] Ochiai A (1957). Zoogeographical studies on the soleoid fishes found in Japan and its neighbouring regions-I. Bull Jpn Soc Scient Fish.

[44] Otsuka Y (1936). The faunal character of the Japanese Pleistocene marine Mollusca, as evidence of climate having become colder during the Pleistocene in Japan. Biogeograph Soc Japan.

[45] Breiman L (1996). Bagging predictors. Mach Learn.

[46] Schapire RE (1990). The strength of weak learnability. Mach Learn.

[47] Breiman L (2001). Random forests. Mach Learn.

[48] Wang Q, Garrity GM, Tiedje JM, Cole JR (2007). Naive Bayesian classifier for rapid assignment of rRNA sequences into the new bacterial taxonomy. Appl Environ Microb.

[49] Bishop CM (1996). Neural networks for pattern recognition.

[50] Cover T, Hart P (1995). Nearest neighbor pattern classification. IEEE Trans Information Theory.

[51] Chang CC, Lin CJ (2011). LIBSVM: a library for support vector machines. ACM Trans Intel Syst Tec (TIST).

[52] Salwinski L, Miller CS, Smith AJ, Pettit FK, Bowie JU, Eisenberg D (2004). The database of interacting proteins: 2004 update. Nucleic Acids Res.

[53] Prasad TSK, Goel R, Kandasamy K, Keerthikumar S, Kumar S, Mathivanan S, Telikicherla D, Raju R, Shafreen B, Venugopal A (2009). Human protein reference Database-2009 update. Nucleic Acids Res.

[54] Guldener U, Munsterkotter M, Oesterheld M, Pagel P, Ruepp A, Mewes HW, Stumpflen V (2006). MPact: the MIPS protein interaction resource on yeast. Nucleic Acids Res.

[55] Guo Y, Li M, Pu X, Li G, Guang X, Xiong W, Li J (2010). PRED_PPI: a server for predicting protein-protein interactions based on sequence data with probability assignment. BMC research notes.

[56] Perovic V, Sumonja N, Gemovic B, Toska E, Roberts SG, Veljkovic N (2017). TRI_tool: a web-tool for prediction of protein-protein interactions in human transcriptional regulation. Bioinformatics.

[57] Zhang JM, Jia K, Jia JM, Qian Y (2018). An improved approach to infer protein-protein interaction based on a hierarchical vector space model. BMC Bioinformatics.

[58] Park Y (2009). Critical assessment of sequence-based protein-protein interaction prediction methods that do not require homologous protein sequences. BMC Bioinformatics.

